# Regression and Complications of z-score-Based Giant Aneurysms in a Dutch Cohort of Kawasaki Disease Patients

**DOI:** 10.1007/s00246-017-1590-0

**Published:** 2017-02-24

**Authors:** S. M. Dietz, I. M. Kuipers, J. C. D. Koole, J. M. P. J. Breur, Z. Fejzic, S. Frerich, M. Dalinghaus, A. A. W. Roest, B. A. Hutten, T. W. Kuijpers

**Affiliations:** 1 Department of Pediatric Hematology, Immunology and Infectious Diseases, Emma Children’s Hospital, Academic Medical Centre (AMC), Meibergdreef 9, 1105 AZ Amsterdam, The Netherlands; 2 Department of Pediatric Cardiology, Emma Children’s Hospital, AMC, Amsterdam, The Netherlands; 3 Department of Pediatric Cardiology, Wilhelmina Children’s Hospital, University Medical Centre Utrecht, Utrecht, The Netherlands; 4 Department of Pediatric Cardiology, Amalia Children’s Hospital, Radboud University Medical Centre, Nijmegen, The Netherlands; 5 Department of Pediatric Cardiology, Academic Hospital Maastricht, Maastricht, The Netherlands; 6 Department of Pediatric Cardiology, Sophia Children’s hospital, Erasmus Medical Centre, Rotterdam, The Netherlands; 7 Department of Pediatric Cardiology, Willem-Alexander Children’s Hospital, Leids University Medical Centre, Leiden, The Netherlands; 8 Department of Clinical Epidemiology, Biostatistics and Bioinformatics, AMC, Amsterdam, The Netherlands

**Keywords:** Mucocutaneous lymph node syndrome (Kawasaki disease), Coronary aneurysms, Major cardiac event, Myocardial infarction

## Abstract

Kawasaki disease (KD) is a pediatric vasculitis. Its main complication is the development of coronary artery aneurysms (CAA), with giant CAA at the end of the spectrum. We evaluated regression and event-free rates in a non-Asian cohort of patients with giant CAA using the current z-scores adjusted for body surface area instead of absolute diameters. KD patients with giant CAA (*z*-score ≥10) visiting our outpatient clinic between January 1999 and September 2015 were included. Patient characteristics and clinical details were extracted from medical records. Regression was defined as all coronary arteries having a z-score of ≤3. A major adverse event was defined as cardiac death, myocardial infarction, cardiogenic shock, or any coronary intervention. Regression-free and event-free rates were calculated using the Kaplan–Meier method. We included 52 patients with giant CAA of which 45 had been monitored since the acute phase. The 1-, 2-, and 5-year regression-free rates were 0.86, 0.78, and 0.65, respectively. The 5-year, 10-year, and 15-year event-free rates were 0.79, 0.75, and 0.65, respectively. Four children, whose CAA would not have been classified as ‘giant’ based on absolute diameters instead of z-scores, had experienced an event during follow-up. Conclusion: We found a high percentage of children in whom the lumen of giant CAA completely normalized. Four children not classified as ‘giant’ based on absolute diameters with z-scores of ≥10 experienced a cardiac event. Hence, the use of z-scores seems to be justified.

**Electronic supplementary material:**

The online version of this article (doi:10.1007/s00246-017-1590-0) contains supplementary material, which is available to authorized users.

## Introduction

Kawasaki disease (KD) is a pediatric vasculitis of the medium-sized arteries [1]. Although its exact origin is still unknown, it is thought to be caused by an infectious agent in genetically predisposed children [2]. While the disease is self-limiting, complications occur, with coronary artery aneurysms (CAA) being the most important one. CAA develop in approximately 25% of all untreated patients, although the introduction of intravenous immunoglobulins (IVIG) as treatment has decreased this percentage substantially [3].

At the extreme end of the spectrum of CAA are giant CAA. In Asian cohorts, giant CAA typically do not regress, but evidence is scarce in the current era of more standardized treatment protocols. Most studies use cut-offs based on absolute diameters instead of *z*-scores. *Z*-scores are preferred as they correct the absolute size in mm for body surface area (BSA) of the child [4].

Persistence of giant CAA causes an increased chance of thrombosis within and perfusion abnormalities distal to the CAA, potentially inducing ischemia and myocardial infarction (MI) [5]. Also, the persistence of giant CAA requires life-long administration of anticoagulation by low-molecular-weight heparin or vitamin K antagonists.

In this study, we report on a cohort of patients with giant CAA based on z-scores, evaluating regression and cardiac complications.

## Methods

### Study Population and Data Collection

The Academic Medical Centre in Amsterdam (The Netherlands) is a tertiary referral center for patients with KD. At our center, we have a multidisciplinary outpatient clinic for cardiologic and/or (immunologic) long-term follow-up. The cohort of KD patients at out center consists of patients who were admitted to our center during the acute phase and who are admitted to other hospitals during the acute phase and are referred to our center for follow-up.

Patients with KD visiting our outpatient clinic between January 1999 and September 2015 were eligible. Patients were included if they had giant CAA during the acute phase of the disease. For analyses purposes, patients who were missed during the acute phase and patients of whom no information of the acute phase was available were excluded.

We retrospectively extracted clinical details from the medical records, i.e., gender, age at disease onset, (in)complete disease presentation, treatment with IVIG including the day of first IVIG treatment, IVIG re-treatment, treatment with steroids, aspirin and/or anticoagulation therapy, and size of the CAA at initial presentation and during follow-up.

### Coronary Artery Aneurysms

The CAA status was taken from information in echocardiography reports. The highest *z*-score of the left main coronary artery, right coronary artery, or left anterior descending artery within the first 6 weeks was chosen in order to specify CAA [4, 6]. Giant CAA are defined by a z-score of ≥10 [4]. When using the Japanese criteria based on absolute diameters, a giant CAA is defined as diameter of ≥8 mm [7].

### Outcomes: Regression and Major Adverse Events

During follow-up, z-scores based on echocardiography, coronary angiographies (CAG’s), Magnetic Resonance Imaging (MRI’s), or Computer Tomography (CT)-scans were calculated. Regression was defined as all coronary arteries having a z-score of ≤3. CAA were also considered to have regressed when only a dilation of the origin of the coronary arteries with a *z*-score of 3 to 3.5 was present.

A major adverse event was defined as cardiac death, MI, cardiac arrest or cardiogenic shock, coronary artery bypass grafting (CABG), or percutaneous coronary intervention (PCI).

### Statistical Analysis

#### Regression

We calculated the number of days until all coronary arteries were regressed as demonstrated by an imaging procedure. If regression did not occur, the follow-up time was calculated until the date of the last imaging procedure with a maximum of 15 years. A regression-free survival curve was then constructed by means of the Kaplan–Meier method.

#### Major Adverse Event

If imaging suggested ischemia or infarction without preceding clinical presentation, the time from diagnosis until the date of the imaging was calculated. Patients were censored if no event occurred; follow-up time was calculated until the last registered imaging procedure with a maximum of 15 years. We assessed the 5-, 10-, and 15-year event-free survival using Kaplan–Meier analysis.

## Results

### Patient Population

In total, 52 patients with giant CAA visited our outpatient clinic. In six patients, KD was missed. These six patients (5 men, 1 woman) presented with cardiac complaints due to ischemia and/or MI based on thrombus formation in a CAA or stenosis proximal or distal to a CAA. None of the patients had signs of atherosclerosis or cardiac disease, making KD the most likely cause of their giant CAA. In one extra patient, no information about the acute phase of the disease was available.

The remaining 45 KD patients had echocardiographic examinations within 6 weeks after acute disease onset. Patient characteristics and clinical data are shown in Table 1. Only 18 (40%) children would have classified as having ‘giant’ CAA based on absolute diameters. All but one child had been treated with IVIG. Most of them were treated with 2 g/kg IVIG in a single gift. Two children received 400 mg/kg during 5 days, and in 4 children dosing had not been recorded. Apart from a second IVIG gift, 11 (25%) children received methylprednisolone, oral prednisone, or both successively. Four (10%) children subsequently received either anakinra (IL-1 inhibitor) or infliximab (TNF-α inhibitor) due to IVIG and corticosteroid resistance. All children received high-dose aspirin during the acute phase followed by the standard low-dose aspirin. Twenty-four children received additional anticoagulants (low-molecular-weight heparin or vitamin K antagonists). Most children not receiving anticoagulants at any point had KD in an earlier era. Three children received an additional platelet aggregation inhibitor.

**Table 1:** Demographic and clinical characteristics of patients with giant CAA during the acute disease

	Giant CAA *n* = 45
Male gender, *n* (%)	41 (91)
Age at disease onset (years)^a^	1.0 (0.3–2.8)
Complete disease, *n* (%)	29 (64)
IVIG treatment, *n* (%)	44 (98)
Day first IVIG treatment+^a^	10 (7–18)
Second IVIG treatment, *n* (%)	19 (42)
Steroid treatment, *n* (%)	11 (24)
Highest-ever *z*-score^a^	17.1 (14.0–24.1)
Highest-ever diameter (mm)^a^	6.9 (5.7–8.5)
Follow-up time (years)^a^	6.9 (2.6–15)

*IVIG* intravenous immunoglobulins

^a^Median (interquartile range), + Day calculated from first day of fever

In 13 (29%) children, aspirin was discontinued after the CAA were considered to have regressed completely. They had received aspirin for a median of 17.3 months (Interquartile range, IQR 8.8-151.5 months). None of these children experienced a cardiac event during or after discontinuation of aspirin therapy.

### Regression of Giant CAA

**Fig. 1:**
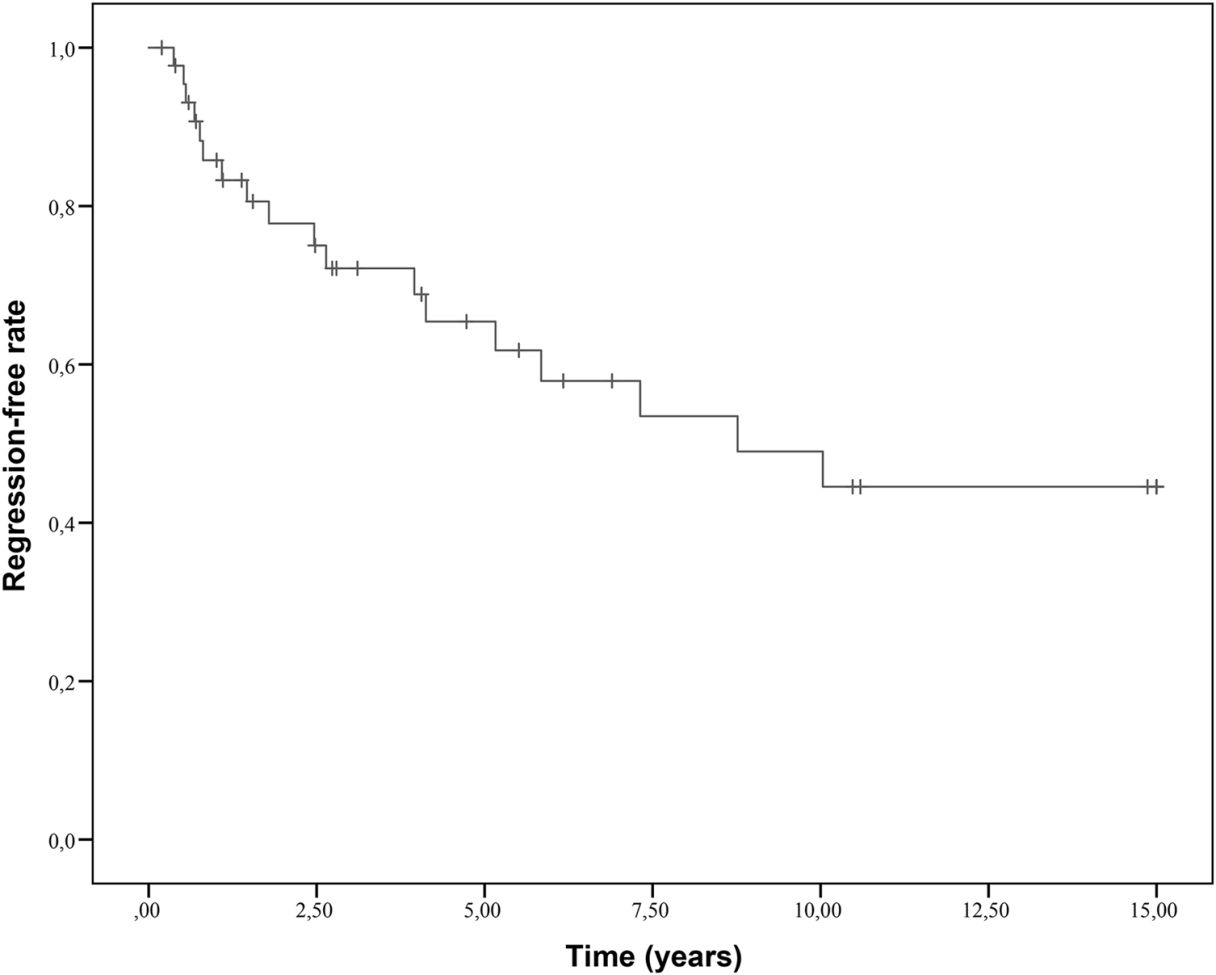
Kaplan–Meier estimates of regression-free survival of patients with giant CAA. + Indicates censored patients

**Fig. 2:**
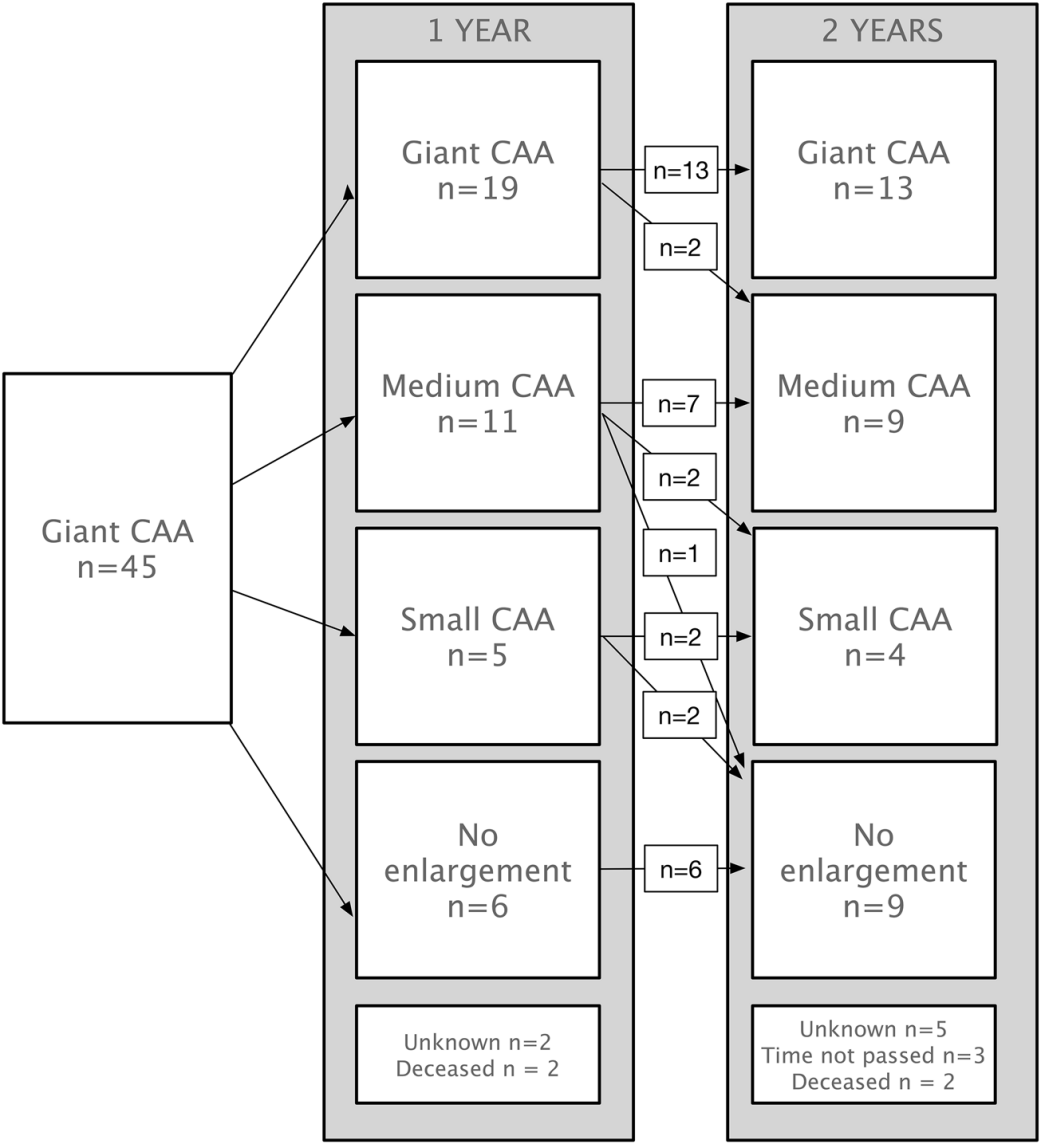
CAA size after 1 and 2 years. *Small CAA z-*score 3–5, *medium CAA z-*score 5–10, *giant CAA* ≥*10. CAA=* coronary artery aneurysms

The 1-, 2-, and 5-year regression-free rates were 0.86, 0.78, and 0.65, respectively (Fig. 1). Remarkably, in 4 children the giant CAA had completely normalized more than 5 years after the acute phase of the disease. Apart from the 9 children with giant CAA going into complete regression, the largest change in z-score was observed in the first 2 years with an additional 13 children showing regression to medium or small CAA (Fig. 2).

Due to the small number of children and the accompanying lack of power, we could not perform multivariable regression analyses to identify predictors for the persistence of giant CAA. Strikingly, only 4 (9%) children were girls, of which 2 had completely regressed CAA. A total of 23 (50%) children were under the age of 1 during the acute disease, of which 4 regressed within the first and an additional 2 within the second year. Of the 22 children over the age of 1 during the acute disease, 2 regressed within a year and 1 other child within the first 2 years.

Of the 19 children receiving a second IVIG dose, 6 had completely regressed CAA in a median time of 2.9 years (IQR 1.3–4.4). Of the 11 children receiving subsequent steroid treatment, 5 had regressed in a median time of 4.0 year (IQR 1.2–4.6).

Of the 14 children with an original z-score of 10–15, 11 (80%) went into complete regression. Of the children with an original z-score of 15–20, only 4 (25%) out of 16, and for the children with an original z-score of > 20, we observed that only 3 (20%) out of 15 completely regressed (Supplemental Fig. 1).

### Major Adverse Event

**Fig. 3:**
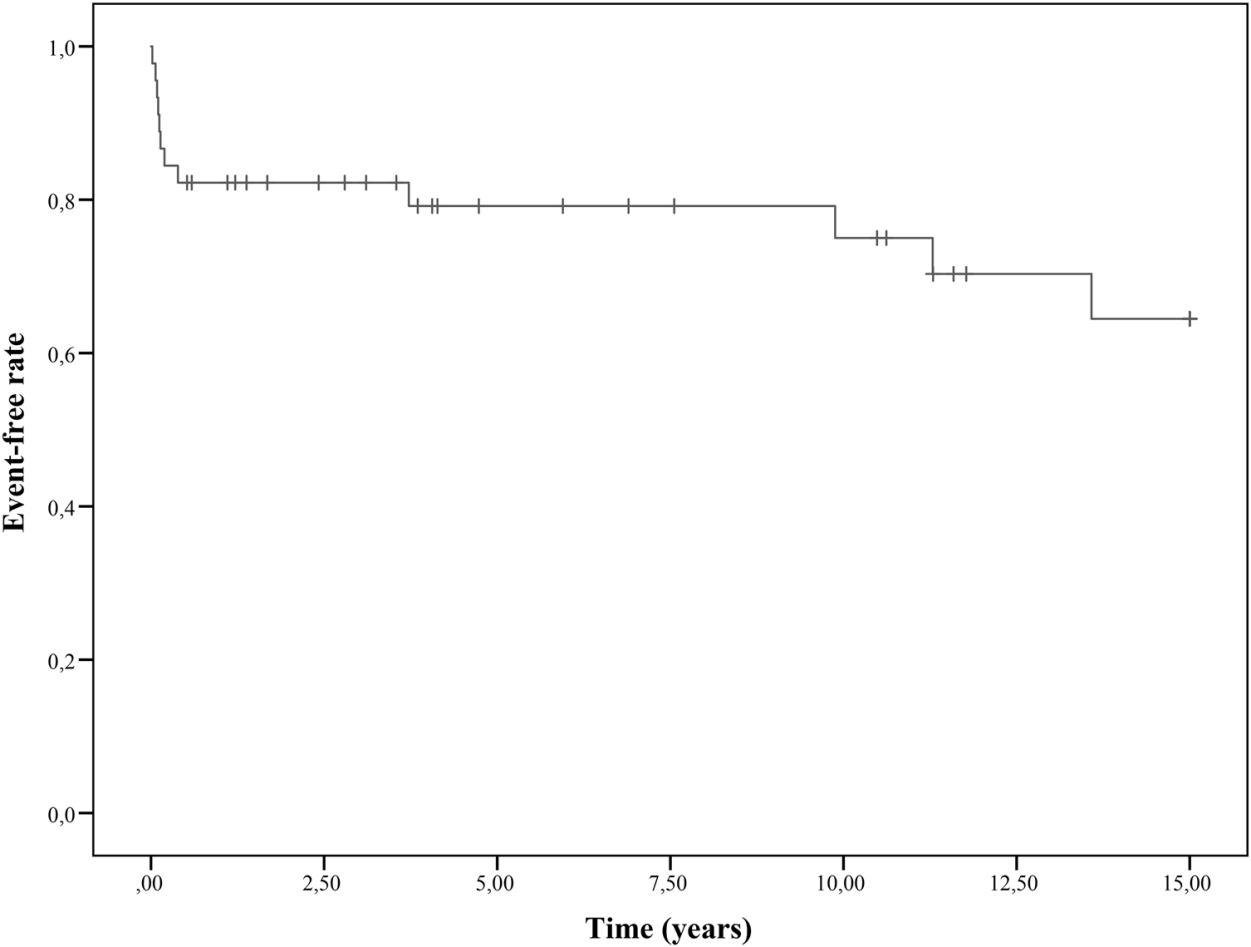
Kaplan–Meier estimates of major adverse event-free survival of patients with giant CAA. Cardiac event- and cardiac intervention-free survival. + Indicates censored patients

A total of 12 cardiac events or interventions took place after a median time of 0.17 years (range 0.02–13.58 years) (Fig. 3). All events accompanied with clinical symptoms occurred within 5 months after the acute disease, and interventions and subclinical events occurred later. All events happened in children with, at that time, non-regressed CAA. The 5-, 10-, and 15-year adverse event-free rates were 0.79, 0.75, and 0.65, respectively. In 4 children who would not have classified for a giant CAA according to the absolute diameters, a serious cardiac event took place, although significantly more events occurred in children with giant CAA based on absolute diameters (*p* = 0.041).

Two children died as a result of MI. Another 6 children experienced MI. At time of the ischemic event, 3 children were using vitamin K inhibitors or low-weight heparin, 3 children were taking aspirin, and in 2 children antiplatelet therapy had not been initiated (yet). In 3 of them, the ischemia was only apparent upon contrast-enhanced imaging of the heart as clinical symptoms and cardiac signs were absent.

Five children had a cardiac arrest or cardiogenic shock. Four children had undergone CABG at a median age of 15.9 years (range 10.5–22.0 years).

Four of the children who experienced MI obtained a cardiac MRI with adenosine stress testing during follow-up, showing hypo- or akinesia of the wall in the infarcted areas but an otherwise good systolic function. Another 15 children obtained a MRI with adenosine stress testing, showing no functional abnormalities.

### Stenosis

In 8 children, coronary arterial stenosis was apparent with subsequent CABG in 3 and a coronary balloon dilation in 1 patient(s). In 2 children, the stenoses became only apparent at autopsy and in 2 children the stenosis did not require immediate intervention. Most stenoses were seen directly proximal or distal to the CAA. None of these children had completely regressed CAA. Five of the children with arterial stenoses had experienced MI. In Table 2, the distribution of patients with cardiac events and with stenosis is shown.

**Table 2:** Distribution of cardiac events and stenosis in patients with giant CAA

	Stenosis + MI	MI	Stenosis	–	Total
Cardiac arrest/shock	2^a^	1	–	2	5
CABG	2	–	1	1	4
–	1	2	2+	–	5
**Total**	5	3	3	3	14

*CAA* coronary artery aneurysms, *CABG* coronary artery bypass grafting

^a^2 children died as a result of MI, + 2 children had stenosis but no event

## Discussion

In a large Dutch cohort of KD patients with giant CAA based on z-scores, visiting our outpatient clinic during a 15-year period, we calculated 1-year, 2-year, and 5-year regression-free rates of 0.86, 0.78, and 0.65. The 5-year, 10-year, and 15-year event-free rates were 0.79, 0.75, and 0.65. This is the first study solely evaluating patients with giant CAA based on z-scores in a Western population.

A study by Chih at al., following 27 children with giant CAA (≥ 8 mm), found that none completely regressed [8]. In a Taiwanese study evaluating KD patients including 27 patients with giant CAA (≥8 mm), the same result was found [9]. On the other hand, a recent study reviewing 90 US patients with giant CAA based on z-scores, found that in 19% of the patient with giant CAA regression occurred [10]. In our study, 18 (40%) children with a z-score of ≥ 10 completely regressed. Of these children, 50 and 78% of the regression occurred within the first 2 and 5 years, respectively. In 4 out of 45 children, the *z*-scores decreased to <3 more than 5 years after disease onset. The decline of the *z*-scores in these children is suggested to be due to the normal growth of these children rather than a decline of the absolute CAA diameter. Our finding that regression mainly takes place in the first 5 years is supported by earlier studies. Lin et al. found a 5-year persistence rate of 46% for medium-sized CAA with little regression after [9]. Kato et al., studying a group of patients with CAA of all sizes, found that 90% of regression occurred within the first 2 years [11].

In a large Japanese study, Tsuda et al., studying 245 patients with giant CAA (≥8 mm), found 10-, 20-, and 30-year cardiac event-free survival rates of 64, 48, and 36%, respectively [5]. The aforementioned study by Chih et al. found 10- and 20-year ischemia-free rates of 52 and 21% [8]. Our study showed higher cardiac event-free rates, which could partly be the result of the use of z-scores instead of absolute diameter cut-off. However, 4 children, whose CAA would not have been classified as ‘giant’ based on absolute diameters, did experience an event (cardiac arrest, MI, and cardiogenic shock), which suggests that *z*-scores are helpful in identifying patients at high risk. Also, all but one patient in our cohort received IVIG, improving outcome as IVIG was found to be an independent risk factor for major adverse cardiac events (MACE) in a recent study [10].

Most events happened within the first months after the acute phase. Yet, in 3 patients, echocardiography or MRI showed signs of a small MI or subclinical ischemia, 10 years or more after the acute disease .Although conventional coronary angiography is still the gold standard to assess coronary anatomy and possible stenosis, this technique is invasive and exposes the child to radiation. Low-dose CT angiography is becoming more widely available and decreased the radiation burden significantly [12]. Using echocardiography or cMRI with additional adenosine stress testing, systolic function and flow reserve capacity can be evaluated, yet cMRI cannot be performed in young children without the use of anesthesia. The results of these 3 patients suggest that all children should be followed up with regular intervals to assess vascular flow reserve capacity using a combination of these techniques as we proposed previously [13].

Although we only observed cardiac events in children with persisting CAA at that time, it is unlikely that regression of CAA to a normal diameter of the arterial lumen will eliminate all future cardiovascular risk. Even if the lumen has a normal diameter, the arterial wall is supposed to be damaged and unable to adequately dilate upon increased cardiac demand [14]. In a recent study using optical coherence tomography, changes in the coronary artery wall structure, especially intimal hyperplasia, were seen in CAA but also in segments where the CAA had regressed [15]. This study indicates that these patients require life-long follow-up, even if the arterial lumen has gone back to normal size. In 13 children of our cohort, aspirin therapy was discontinued after the CAA were considered to have regressed. Regarding the remodeling, persistent damage, and increased stiffness of the arterial wall years after the acute phase, it is questionable whether antiplatelet medication should ever be discontinued in patients with regressed giant CAA. However, none of the children in whom aspirin had been discontinued experienced an event. This is in concordance with the study by Friedman et al. who found that none of the patients with regressed CAA experienced MACE [10]. Stenosis, a result of a remodeling process of the artery, only occurred in children with CAA that had not completely regressed. In summary, more research is necessary for definite recommendations regarding (life-long) aspirin therapy.

### Limitations

We calculated z-scores from absolute diameters described in echocardiography, CAG-, MRI-, or CT-reports. Since approximately half of the patients were admitted to other hospitals during the acute phase of the disease, pediatric cardiologists in other centers had generated many of the early echocardiographies. Echocardiography is known for its measurement uncertainty, which could have influenced the regression as well as the time-to-regression.

For the time-to-regression, we registered the time until the first imaging procedure that demonstrated normality of all coronary arteries. The precise time of regression can therefore not exactly be defined. Hence, the time-to-regression has to be considered as the maximum time in which regression took place.

As this was a retrospective study, imaging was not performed according to a set protocol. This means subclinical ischemia could have been missed in children if no suitable imaging technique was performed.

## Conclusion

In a Dutch cohort of KD patients with giant CAA based on z-scores followed up from the acute phase, the 1-, 2-, and 5-year regression-free rates were 0.86, 0.78, and 0.65. The 5-, 10-, and 15-year major adverse event-free rates were 0.79, 0.75, and 0.65, respectively. In 4 children whose CAA would not have classified as being ‘giant’ according to absolute diameters instead of z-scores, a cardiac event took place. Therefore, z-scores are suggested to be a more sensitive tool to decide on life-long regular follow-up of KD children.

## Electronic supplementary material

Below is the link to the electronic supplementary material.

Supplementary material 1 (DOCX 21 KB)
