# Supplementary material for: Regression and Complications of z-score-Based Giant Aneurysms in a Dutch Cohort of Kawasaki Disease Patients
Source: Pediatr Cardiol. 2017 Feb 24;38(4):833–9. doi: 10.1007/s00246-017-1590-0 (PMC5388726; doi:10.1007/s00246-017-1590-0)
Supplement: Supplementary file 1 — Supplementary material 1 (DOCX 21 KB) [file 246_2017_1590_MOESM1_ESM.docx]

**Supplemental figure 1:** **Kaplan-Meier estimates of regression-free survival of patients with giant CAA, subdivided based on highest-ever z-score.**

*Footnote: (+) indicates censored patients.*
